# Performance of healthy persons under pain in different cognitive load tasks: An event‐related potential study on experimental pain individuals

**DOI:** 10.1002/brb3.1713

**Published:** 2020-06-18

**Authors:** Kangling Wang, Guiyuan Cai, Shimin Huang, Yuqi Li, Rongdong Li, Wen Wu

**Affiliations:** ^1^ Department of Rehabilitation Medicine Zhujiang Hospital Southern Medical University Guangzhou China

**Keywords:** cognitive load, electroencephalogram, event‐related potentials, oscillation, pain

## Abstract

**Objective:**

This study aims to determine how brain activities underlying task with different cognitive load would be modulated by the painful state using electroencephalography.

**Methods:**

The pain state was established by spraying capsaicin on subjects’ left inner forearm. A total of 20 experimental pain subjects and 20 matched nonpain controls underwent cognitive tasks with electroencephalogram recording. We collected and analyzed behavioral and event‐related potential (ERP) data.

**Results:**

High cognitive tasks exhibited significantly longer response times and lower accuracies than low‐load tasks. The experimental pain group displayed a significantly lower accuracy than the control group. In addition, the experimental pain group showed no significance between high and low cognitive tasks in early ERP components (amplitude of N1, P2, N2, and early part of late positive potential), whereas the control group exhibited significance between different load tasks. Furthermore, we observed a delay peak energy for delta and theta oscillation in Fz 500–800 ms after the onset for pain persons and high cognitive load tasks.

**Conclusions:**

Inadequate early attention modulation, along with delayed peak energy for brain oscillation (delta and theta), could be accountable for a worse performance in cognitive tasks in the experimental pain group. Thus, cognitive load is a highly considerable factor. Overall, this study offers more insights into how healthy population works with cognitive tasks under pain neurologically.

## Graphical abstract

Insufficient early attention modulation combined with a delay peak energy for brain oscillation (delta and theta) lately might be accountable for a worse performance in cognitive task for experimental pain persons. Cognitive load was a highly considerable factor.

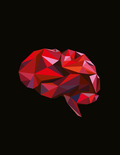

## 1 INTRODUCTION

The interactive correlation between pain and attention in pain population has long been highlighted. Behaviorally, while pain interferes with attention, resulting in poorer performance in tasks, attention can modify pain (Bantick et al., 2002; Sturgeon et al., 2015; Valet et al., 2004). Regarding attention, cognitive load is a potential factor. Studies using experimental pain models have usually supported higher effects on complex dual‐task performance compared with more simple tasks (Moore, Eccleston, & Keogh, 2017), and that only moderate or high levels of cognitive load suppress subjective pain ratings (Romero, Straube, Nitsch, Miltner, & Weiss, 2013; Sturgeon et al., 2015). In addition, fMRI studies have mostly contributed understanding the neural mechanism. Reportedly, overlapping cognitive resources play a role in both pain processing and executive functions (Bantick et al., 2002; Seminowicz & Davis, 2007; Valet et al., 2004; Wiech et al., 2005). Some studies have also reported inhabitation of cognitive tasks on the upregulated and downregulated pain pathway (Bantick et al., 2002; Bushnell, Ceko, & Low, 2013; Kucyi, Salomons, & Davis, 2013; Valet et al., 2004). Studies using event‐related potentials (ERPs) have provided evidence from the temporal aspect. Moreover, some studies have demonstrated that pain affects early components, such as P3 and equivocation of ERP waves, based on the task difficulty, thereby depicting a steal of attentional resources by pain (Folstein & Van Petten, 2008; Houlihan et al., 2004; Samartin‐Veiga, González‐Villar, & Carrillo‐de‐la‐Peña, 2019; Seminowicz & Davis, 2007). Furthermore, in a study, patients with pain did not exhibit decreased amplitudes with increasing task load (Veldhuijzen et al., 2006). Nevertheless, the current temporal evidence is insufficient to assess neural activities that underlie pain and/or cognitive tasks for healthy population.

Specifically, this study aims to investigate the neural activities of experimental pain subjects under cognitive tasks by synchronously recording the cognitive process using electroencephalography (EEG), which can capture the intrinsic brain activity as neural oscillations in different rhythms, as well as ERP, and present the electrophysiological changes in a time domain during the process. We applied two types of pain interferences (chemical heat pain stimulus and sensory pain words) and two types of cognitive loads (high and low). Furthermore, the chemical heat pain stimulus, a definite pain stimulator, was used to simulate the perception of thermal pain, while the sensory pain words served as a potential pain stimulator to distract attention.

The specific hypotheses of this study are as follows: (a) pain stimulus, either definite or potential, or both, interrupt participants’ speed and/or accuracy in behavior (H1); (b) behavioral performance could differ based on different cognitive tasks—the higher the load, the worse the reaction (H2). ERP results would reveal the underlying neural mechanism.

## 2 MATERIALS AND METHODS

### 2.1 Participants

We enrolled 40 healthy students (20 males and 20 females; age: 18–27 years) from the University of Southern Medical University. The inclusion criteria were as follows: right‐handed; fluency in Chinese; normal and corrected‐to‐normal vision. The exclusion criteria were as follows: experiencing any form of pain; been diagnosed or receiving treatment for a psychiatric disorder currently or within the past 5 years; taking any psychotropic or analgesic medication regularly. The study protocol was approved by Ethics Committee of Zhujiang Hospital, Southern Medical University, and we obtained written informed consent from all participants before commencing the study. Before the experiment, all participants self‐valued on the visual analogue scale (VAS). In addition, Anxiety and Depression Scale (ADS; divided into anxiety and depression) and State‐Trait Anxiety Inventory (STAI; divided into state of anxiety and trait of anxiety) were performed after the experiment to not affect subjects’ mood while testing.

### 2.2 Measures and procedure

The experiment was designed for 2 (cognitive load: two‐digit and six‐digit) × 2 (interfering words: pain words and nonpain words comprised neutral, positive, and negative words) × 2 (group: control and experimental pain subjects) conditions. The cognitive load comprised a high load and a low load, distinguished by the length of the string of numbers to be memorized (6 for high and 2 for low cognitive load, respectively). All 400 digits were created by a random number generator, with each type of digits created 50%, respectively. Interfering words included sensory pain words and nonpain words. For pain words, we selected 25 sensory pain words (i.e., throbbing) from the McGil Pain Scale (Chinese version). For nonpain words, we selected 25 neutral, positive, and negative words, respectively, from Chinese Affective Words System (CAWS; Zhang et al., 2014), with low‐level features (i.e., valence, arousal ratings, dominance, and familiarity) of each type of phrases matched. As the number of pain words in the McGil Pain Scale is inadequate to supply sufficient stimulation, we presented all 25 words of each group in four different colors—red, yellow, blue, and green—resulting in 100 words in pain word category and 300 in nonpain word category, to make every word a new stimulation. In addition, color saturation and brightness of red, yellow, blue, and green were matched to eliminate errors between different colors. Both words and digital loads were combined randomly. All participants were randomly divided into two groups. While subjects in the experimental pain group were sprayed 10% capsaicin paste (Professional Arts Pharmacy) on the left inner forearm and covered with plastic wrap, the control group was sprayed on pure water and covered with plastic wrap. During task breaks, a simple VAS was used to score the pain intensity they experienced. If a person's pain perception was lower than 4/10 in the VAS, capsaicin was resprayed on the left inner forearm to maintain a higher than 4/10 pain perception.

**FIGURE 1:**
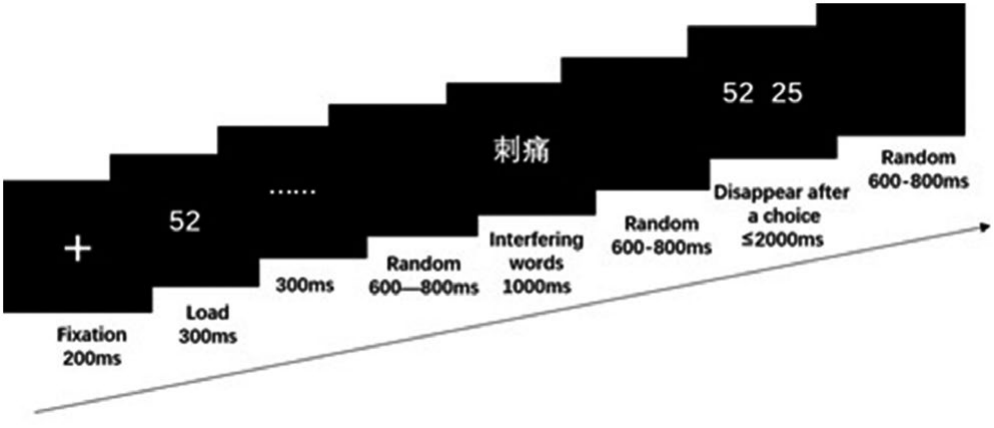
A trial for cognitive tasks under low load

For experimental programming, we used E‐Prime 3.0 software. Every presentation of a trial began with a fixation point “+,” lasting for 200 ms, followed by a sequence of digital load (duration: 300 ms), a “......” screen to maintain the width of attention (duration: 300 ms), empty screen (random time presentation: 600–800 ms), word interference (1,000 ms), empty (black) screen (random time presentation: 600–800 ms), and number selection screen, which comprised two same load numbers with only one of them shown previously. In this screen, participants were asked to select as fast and correctly as possible if the number appeared previously were on the left‐hand side (left index finger pressing key “F” correspondingly) or the right‐hand side (right index finger pressing key “J” correspondingly). The screen disappeared when a button was pressed or within 2,000 ms, after which a finally empty screen (random time presentation: 600–800 ms) closed a trial (Figure 1).

Before the experimental task, subjects conducted 24 practical trials to familiarize themselves with the task. As more trials can enhance the reliability of ERP research, subjects had to conduct a total of 400 trials (practical trials excluded), which were categorized into eight blocks with 50 trials per block. Notably, the interval between two blocks was 2 min; it costed approximately 40–60 min to complete the whole experiment.

### 2.3 EEG recording

Seated in an anatomic chair in a quiet, sound‐insulated, and dark light room, participants were positioned with a 17‐inch computer screen displaying all the stimulus in 1,024 × 798 display size just 60 cm before. The room temperature was maintained at 25°C. All participants were instructed to not use any analgesic, psychotropic substances, or any other medicines 24 hr before the experiment, which could affect the central nervous system. Besides, all participants were requested to take a sound sleep the night before.

We recorded the electrophysiological signals using a 32‐channel cap based on the International 10‐20 System (Biosemi), referred to linked mastoids and grounded at AFz. In addition, vertical electrooculogram recordings were obtained using electrodes placed above and below the left eye, while horizontal eye movements were recorded using electrodes placed over the outer canthus of both eyes. Furthermore, EEG signals were filtered using a 0.05–100 Hz; the sampling rate was 512 Hz; and all interelectrode impedances were kept below 5 kΩ.

### 2.4 Statistics analysis

In this study, E‐Prime 3.0 software was used to extract indexes as response time (RT) and accuracy (AC). Of note, only the trials that had right response were selected. A repeated‐measure ANOVA was performed using SPSS 20 (IBM).

We selected and analyzed night electrodes (F3, Fz, F4, C3, Cz, C4, P3, Pz, and P4) according to the frontal, central to partial, left, and central to right, as reported elsewhere (Cheng, Jiao, Luo, & Cui, 2017; Mu & Han, 2013), As we primarily examined the number selection phase here, the screen of number selection was taken as the stimulus onset, with the former 500 ms and the later 1,000 ms considered a range analyzed. In addition, the 200 ms waveform before zero point was considered the baseline. We performed ERP processing using MATLAB R2013a (MATLAB, RRID:SCR_001622) and EEGLAB 12.0 (EEGLAB, RRID:SCR_007292). After decreasing the sampling rate to 500 Hz, the data were filtered through 0.1–40 Hz.

Continuous variables were segmented into epochs mentioned above. Then, we performed an independent component analysis (ICA) to eliminate EOG after eliminating bad epochs and interpolation of electrodes with high noise. Notable, epochs with correct response were selected for averaging. Next, time–frequency representations of power were evaluated using the short‐time Fourier transform for correct trials and all channels; −500 to 0 ms was baseline. In this study, all statistical analyses were performed in the following frequency band: delta (1–3 Hz), theta (4–7 Hz), alpha (8–13 Hz), beta (13–30 Hz), and gamma (30–50 Hz). Furthermore, the main effects and interactions were statistically analyzed using a repeated‐measure ANOVA for power.

## 3 RESULTS

### 3.1 Demographic statistics

**FIGURE 2:**
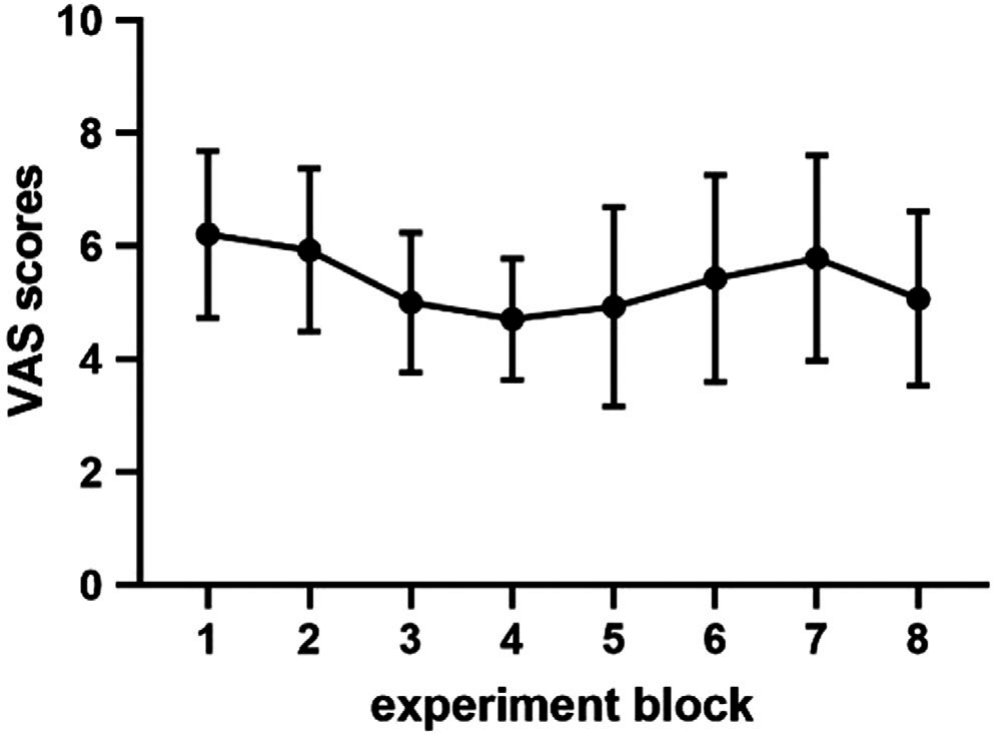
Visual analogue scale (VAS) for different experiment blocks

Table 1 presents the demographic characteristics of both groups. The findings revealed that both groups exhibited no significant difference in age, gender radio, years of education, HADS values, and STAI values. Figure 2 shows the VAS scores in each block, revealing that pain perception in our experiment was steady and mild.

**TABLE 1:** Demographic characteristics and results of *t* tests and chi‐square comparisons between groups (*M* ± *SD*)

	EP *N* = 20	CT *N* = 20	*t*,* X* ^2^	*p*
Age (years)	22.54 ± 2.99	21.69 ± 1.89	0.745	.397
Subject number (female)	20 (11)	20 (9)	0.133	.715
Years of education	16.31 ± 2.25	15.77 ± 1.48	0.519	.478
VAS value	5.38 ± 1.58	—	—	—
HADS				
A	3.46 ± 2.40	3.08 ± 2.29	0.175	.680
D	4.00 ± 2.16	2.31 ± 2.14	4.033	.056
STAI				
S	35.76 ± 7.49	31.69 ± 8.04	1.792	.193
T	35.15 ± 7.35	31.78 ± 9.41	1.045	.317

Abbreviations: A, anxiety; CT, control persons; D, depression; EP, experimental pain persons; HADS, Hospital Anxiety and Depression Scale; S, state of anxiety; STAI, State‐Trait Anxiety Inventory; T, trait of anxiety; VAS, visual analogue assessment scale.

### 3.2 Behavior

**FIGURE 3:**
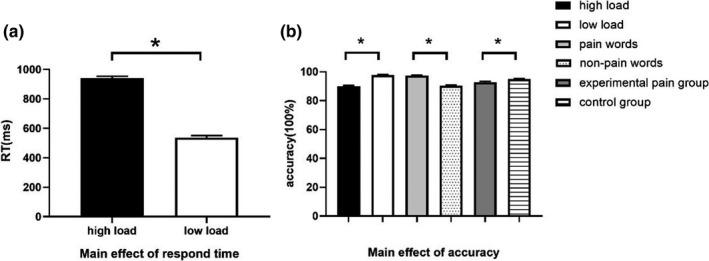
Behavioral results. A. Response time (RT) was longer under high cognitive load compared with low cognitive load (940.31 ± 13.46 ms vs. 538.14 ± 13.46 ms; *F* = 446.512; *p* < .001). B. Accuracy (AC) was higher in low cognitive load than that in high cognitive load (97.70 ± 0.45% vs. 90.06 ± 045%; *F* = 143.085; *p* < .001), higher in pain words than that in nonpain words (97.34 ± 0.45% vs. 90.41 ± 0.45%; *F* = 117.588; *p* < .001), and higher in the control group than that in the experimental pain group (94.93 ± 0.37% vs. 92.83 ± 0.52%; *F* = 10.753; *p* = .001). “*” Significant difference

We performed 2 × 2 × 2 repeated‐measure ANOVA (group × load × word) for RT and AC, respectively, with word as the within‐group factor while group and load as between‐group factors. For both RT and AC, the main effect of cognitive load was statistically significant, demonstrating a well‐differentiated effect of load in this study. We noted no other main effect or interactive effect on RT. Furthermore, main effects of interfering words and groups were found in AC but no interactive effect (Figure 3).

### 3.3 ERP components

**FIGURE 4:**
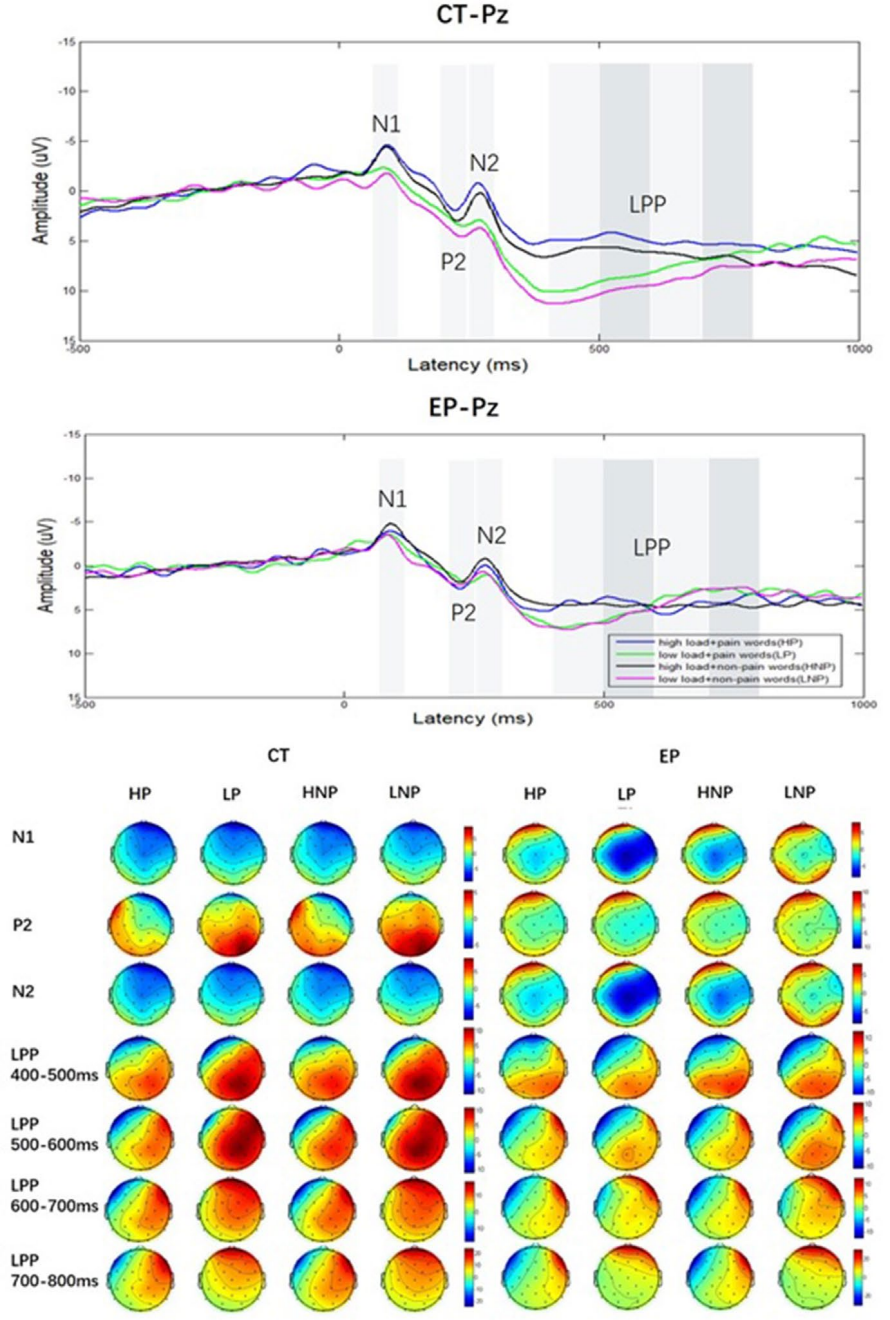
Grand‐averaged event‐related potentials (ERPs) at Pz for different groups under different conditions (CT, control group; EP, experimental pain group)

Time windows for assessing ERP peaks were determined by examining the grand‐averaged waveforms, which were as follows: N1, 72–112 ms; P2, 210–250 ms; and N2, 270–290 ms (Figure 4). As the late positive potential (LPP) tends to reach the maximum amplitude of 400–800 ms after onset (Garland, Froeliger, & Howard, 2015), we divided it into four parts as follows: 400–500, 500–600, 600–700, and 700–800 ms. In addition, we performed 2 × 2×2 × 3 (group × load × word × position) ANOVA of amplitudes for peaks or pieces, respectively, with word as the within‐group factor while group, load, and position as between‐group factors.

Table 2 presents the statistical results of N1, P2, and N2. We constantly observed the significant main effect of position in N1, P2, and N2, which could be related to the nature of our experimental paradigm; however, no significance was found in the interactive effect of group × position. Another constant finding was significant interactive effects of group × load in N1, P2, and N2. Further simple analysis revealed a significance between loads only in the control group. Meanwhile, a significance between groups was noted only in low‐load tasks. Of note, no other interactive effect was found statistically significant.

**TABLE 2:** Statistical results of N1, P2, and N2

Components	Main effect	*F*	Sig	Differences (㎶)	*F*	Sig	Differences (㎶)
N1	Group	2.524	.113	EP: −4.327 ± 0.150 CT: −3.990 ± 0.150			
	**Load**	**21.976**	**.000**	**High: −4.656 ± 0.150** **Low: −3.661 ± 0.150**			
	Word	0.099	.754	Pain words: −4.192 ± 0.150 Nonpain words: −4.125 ± 0.150			
	**Position**	**23.564**	**.000**	**Frontal: −5.620 ± 0.318** **Central: −4.464 ± 0.318** **Parietal: −2.391 ± 0.318**			

	Interaction effect	*F*	Sig	Simple effect			
	Group*load	**15.406**	**.000**	**CT**	**32.299**	**.000**	**High: −4.904 ± 0.227** **Low: −3.076 ± 0.227**
				EP	0.253	.615	High: −4.408 ± 0.227
							Low: −4.246 ± 0.227
				High load	2.377	.123	EP: −4.408 ± 0.227 CT: −4.904 ± 0.227
				**Low load**	**13.236**	**.000**	**EP: −4.246 ± 0.227** **CT: −3.076 ± 0.227**

	Main effect	*F*	Sig	Differences (㎶)			
P2	**Group**	**8.356**	**.004**	**EP: −1.047 ± 0.195** **CT: 1.872 ± 0.195**			
	Load	2.779	.096	High: 1.234 ± 0.195 Low: 1.703 ± 0.195			
	Word	1.863	.173	Pain words: 1.285 ± 0.195 Nonpain words: 1.661 ± 0.195			
	**Position**	**9.040**	**.000**	**Frontal: 0.271 ± 0.414** **Central: 1.325 ± 0.414** **Parietal: 2.823 ± 0.414**			

	Interaction effect	*F*	Sig	Simple effect			
	Group*load	**7.330**	**.009**	**CT**	**9.069**	**.003**	**High: 1.268 ± 0.283** **Low: 2.475 ± 0.283**
				EP	0.513	.474	High: 1.218 ± 0.283
							Low: 0.931 ± 0.283
				High load	0.016	.900	EP: 1.218 ± 0.283 CT: 1.268 ± 0.283
				**Low load**	**14.851**	**.000**	**EP: 0.931 ± 0.283** **CT: 2.475 ± 0.283**

	Main effect	*F*	Sig	Differences (㎶)			
N2	**Group**	**35.184**	**.000**	**EP: −1.538 ± 0.197** **CT: 0.114 ± 0.197**			
	**Load**	**26.507**	**.000**	**High: −1.429 ± 0.197** **Low: 0.005 ± 0.197**			
	Word	0.519	.472	Pain words: −0.813 ± 0.197 Nonpain words: −0.612 ± 0.197			
	**Position**	**32.569**	**.000**	**Frontal: −3.108 ± 0.418** **Central: −1.148 ± 0.418** **Parietal: 2.119 ± 0.418**			

	Interaction effect	*F*	Sig	Simple effect			
	Group*load	**13.080**	**.000**	**CT**	**32.299**	**.000**	**High: −1.107 ± 0.314** **Low: 1.335 ± 0.314**
				EP	0.253	.337	High: −1.752 ± 0.314
							Low: −1.325 ± 0.314
				High load	2.106	.147	EP: −1.752 ± 0.314 CT: −1.107 ± 0.314
				**Low load**	**35.824**	**.000**	**EP: −1.325 ± 0.314** **CT: 1.335 ± 0.314**

Note

Bold fonts indicate statistical differences.

Abbreviations: CT, control group; EP, experimental pain group.

Table 3 presents the statistical results of LPP. In the time domain of 400–500 ms, we found a significant interactive effect of group × load. Further simple analysis revealed a significance between loads only in the control group. Meanwhile, we observed a significance between groups in both high‐ and low‐load tasks. In all time domains, the main effects of group, load, and position were significant. In addition, we noted a consistent phenomenon that the amplitudes were higher in the control group compared with the experimental pain group, higher in low‐load tasks compared with high‐load tasks, and highest in the right hemisphere, followed by that in the central part and left hemisphere.

**TABLE 3:** Statistical results of LPP

Component	Main effect	*F*	Sig	Differences (㎶)	*F*	Sig	Differences (㎶)
LPP (400−500 ms)	**Group**	**72.955**	**.000**	**EP: 1.800 ± 0.250** **CT: 4.822 ± 0.250**			
	**Load**	**21.724**	**.000**	**High: 2.487 ± 0.250** **Low: 4.136 ± 0.250**			
	Word	3.055	.081	Pain words: 3.002 ± 0.250 Nonpain words: 3.620 ± 0.250			
	**Position**	**34.066**	**.000**	**Frontal: 0.559 ± 0.532** **Central: 3.815 ± 0.532** **Parietal: 7.057 ± 0.532**			
		**22.914**	**.000**	**Left: 1.943 ± 0.490** **Central: 2.891 ± 0.490** **Right: 4.568 ± 0.490**			

	Interaction effect	*F*	Sig	Simple effect			
	Group*load	**13.087**	**.000**	**CT**	**27.173**	**.000**	**High: 3.358 ± 0.397** **Low: 6.286 ± 0.397**
				EP	0.432	.511	High: 1.616 ± 0.397
							Low: 1.985 ± 0.397
				**High load**	**9.613**	**.002**	**EP: 1.616 ± 0.397** **CT: 3.358 ± 0.397**
				**Low load**	**58.618**	**.000**	**EP: 1.985 ± 0.397** **CT: 6.286 ± 0.397**

	Main effect	*F*	Sig	Differences (㎶)			
LPP (500−600 ms)	**Group**	**21.113**	**.000**	**EP: 1.828 ± 0.486** **CT: 4.988 ± 0.486**			
	**Load**	**8.521**	**.004**	**High: 2.404 ± 0.486** **Low: 4.411 ± 0.486**			
	Word	0.915	.340	Pain words: 3.079 ± 0.486 Nonpain words: 3.737 ± 0.486			
	**Position**	**13.715**	**.000**	**Frontal: 1.033 ± 0.595** **Central: 3.800 ± 0.595** **Parietal: 5.395 ± 0.595**			
		**73.074**	**.000**	**Left: 0.864 ± 0.582** **Central: 3.785 ± 0.582** **Right: 5.575 ± 0.582**			

	Interaction effect	*F*	Sig	Simple effect			
	group*load	1.240	.130	—	—	—	

	Main effect	*F*	Sig	Differences (㎶)			
LPP (600−700 ms)	**Group**	**21.849**	**.000**	**EP: 2.428 ± 0.591** **CT: 6.337 ± 0.591**			
	**Load**	**11.280**	**.001**	**High: 2.978 ± 0.591** **Low: 5.786 ± 0.591**			
	Word	0.819	.366	Pain words: 4.004 ± 0.591 Nonpain words: 4.760 ± 0.591			
	Position	0.908	.405	Frontal: 3.600 ± 0.724 Central: 4.639 ± 0.724 Parietal: 4.907 ± 0.724			
		**87.879**	**.000**	**Left: 1.443 ± 0.693** **Central: 4.761 ± 0.693** **Right: 6.943 ± 0.693**			

	Interaction effect	*F*	Sig	Simple effect			
	group*load	0.778	.460	—	—	—	

	Main effect	*F*	Sig	Differences (㎶)			
LPP (700−800 ms)	**Group**	**16.700**	**.000**	**EP: 3.653 ± 0.596** **CT: 7.096 ± 0.596**			
	**Load**	**21.786**	**.000**	**High: 3.409 ± 0.596** **Low: 7.340 ± 0.596**			
	Word	0.616	.433	Pain words: 5.044 ± 0.596 Nonpain words: 5.705 ± 0.596			
	Position	1.666	.191	Frontal: 6.366 ± 0.729 Central: 5.264 ± 0.729 Parietal :4.493 ± 0.729			
		**94.816**	**.000**	**Left: 2.321 ± 0.693** **Central: 5.709 ± 0.693** **Right: 8.093 ± 0.693**			

	Interaction effect	*F*	Sig	Simple effect			
	Group*load	0.313	.576	—	—	—	

Note

Bold fonts indicate statistical differences.

Abbreviations: CT, control group; EP, experimental pain group.

### 3.4 Time and frequency

As group and load were the key significant factors but not interfering words in ERP, we further examined oscillation based on the group and load, by dividing and comparing among groups following the combination of group and load (control group with high load [CTH], control group with low load [CTL], experimental pain group with high load [EPH], and experimental pain group with low load [EPL]). We performed 4 × 3 (group [CTH, CTL, EPH, and EPL] × position) ANOVA for every time point (every 100 ms, 0–1,000 ms after onset) and every frequent band.

**FIGURE 5:**
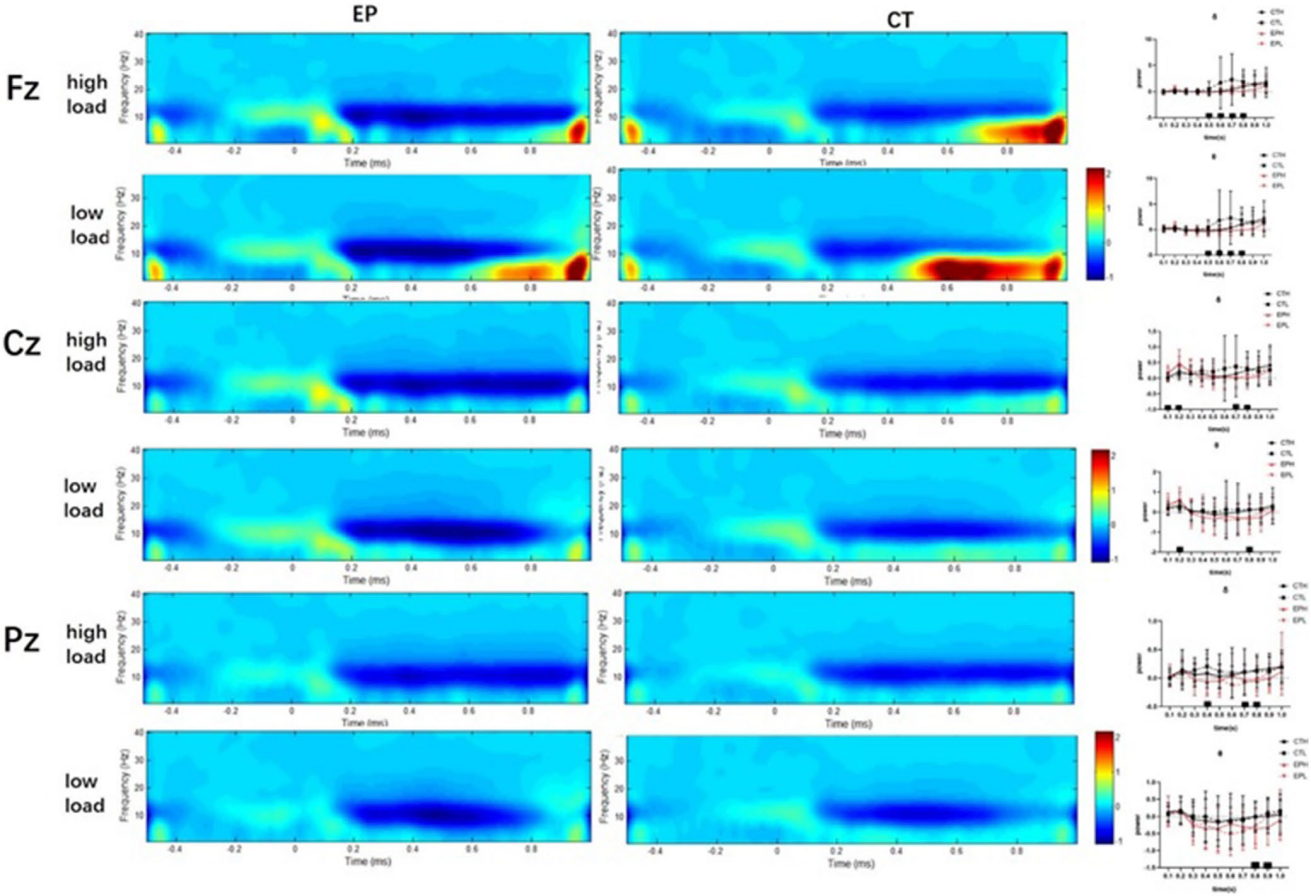
The time–frequency decomposition of the electroencephalogram (EEG) at Fz, Cz, and Pz. (The left side of the maps, name of the site. EP, experimental pain subjects; CT, controls. The right side of the maps, Gantt charts with a statistical difference between groups based on timing. Of note, Fz had the most significant difference and a change in trend according to condition.)

Table 4 presents the statistical results of frequencies. Figure 5 shows the time–frequency decomposition at Fz, Cz, and Pz. We found the constant main effect of position and group in delta and theta in a later time domain but not in alpha, beta, or gamma, indicating a delay peak for delta and theta under pain and in high cognitive load tasks.

**TABLE 4:** Statistical results of frequencies

Frequency	Time (ms)	Main effect		Frequency	Time (ms)	Main effect	
		Group (*F*/Sig.)	Position (*F*/Sig.)			Group (*F*/Sig.)	Position (*F*/Sig.)
Delta	100	1.391/.252	1.672/.195	Theta	100	1.616/.202	1.511/.227
	200	0.578/.450	1.042/.358		200	3.019/.055	1.332/.270
	300	0.422/.518	0.909/.407		300	3.208/.060	0.591/.556
	400	2.198/.142	1.005/.371		400	0.018/.893	0.370/.692
	500	**3.813/.029**	**3.963/.023**		500	**6.200/.004**	0.986/.378
	600	**4.042/.045**	**3.342/.041**		600	**4.491/.032**	**3.586/.047**
	700	**6.848/.006**	**37.806/.001**		700	**7.425/.004**	**7.437/.001**
	800	**5.460/.006**	**17.325/.000**		800	**12.218/.000**	**3.933/.045**
	900	1.178/.309	**21.861/.000**		900	2.386/.127	**18.263/.000**
	1,000	1.356/.260	**27.956/.000**		1,000	1.562/.210	**28.149/.000**
Alpha	100	1.135/.316	2.142/.125	Beta	100	2.841/.054	1.069/.348
	200	0.154/.811	0.140/.869		200	2.360/.118	0.061/.941
	300	**3.841/.044**	0.032/.968		300	**5.783/.012**	0.133/.875
	400	2.391/.121	0.016/.985		400	**5.682/.012**	0.139/.871
	500	3.172/.055	0.117/.890		500	0.561/.456	0.574/.565
	600	**4.658/.017**	0.819/.445		600	0.710/.402	1.461/.238
	700	**5.656/.007**	1.693/.191		700	0.563/.572	1.602/.208
	800	0.069/.794	2.528/.087		800	0.681/.509	1.428/.246
	900	3.290/.074	2.694/.079		900	0.619/.541	1.651/.199
	1,000	**6.179/.000**	**12.691/.000**		1,000	0.807/.450	**1.326/.001**
Gamma	100	1.282/.108	0.211/.810				
	200	**13.324/.000**	1.234/.066				
	300	3.155/.080	**4.124/.020**				
	400	2.217/.116	1.838/.166				
	500	1.815/.097	1.572/.214				
	600	0.647/.424	0.843/.434				
	700	3.204/.078	0.926/.401				
	800	1.936/.076	1.267/.288				
	900	2.564/.080	0.482/.619				
	1,000	1.423/.240	**6.925/.002**				

Note

Bold fonts indicate statistical differences.

## 4 DISCUSSION

This study included two factors (pain stimulus and cognitive loads) that we believed could affect a person's cognitive process. Countering our original hypothesis, behavioral results (AC) revealed a well‐differentiated effect on all these factors, and ERP results revealed no significant difference in potential pain stimulus (interfering words) but definite pain stimulus and cognitive loads. Unlike overall and distinct interactive effect of load and stimulus in the control group, the experimental pain group exhibited no difference between loads. Furthermore, the time–frequency analysis supported a later oscillational difference of delta and theta, adding further evidence for neural activity.

### 4.1 Potential pain stimulus (pain words) as a warning sign

Previously, we hypothesized that pain subjects could be easily attracted by pain words unconsciously, thereby resulting in worse behavioral results. Remarkably, tasks related to interfering pain words had a higher correct rate than nonpain words, although ERP components revealed no difference between words. Patients with chronic pain who are inadaptable to pain stimulus and are gradually accompanied by emotional symptoms like depression and anxiety could, thus, have attention bias; they can easily be indulged in pain‐related stimulus and report difficulty in relieving from them. Conversely, the response of healthy people to pain stimuli is adaptive and alerting. Of note, attention‐seeking nature of pain is essential for individual survival, diverting attention immediately from likely harm and urging people to seek out for good and safety. As we mainly recruited healthy persons as subjects, potential pain stimulus acted more like a warning sign than an interfering factor. Hence, alertness to pain led to more accurate responses to pain word‐related tasks.

### 4.2 Definite pain stimulus (chemical heat stimulus), cognitive load, and ERP responses

#### 4.2.1 ERP components revealed a deficit in attention modification in pain subjects

In this study, definite pain stimulus mimicked a moderate pain perception, with the VAS score of about 5. It is broadly agreed that attentional‐demanding activities can decrease pain intensity scores in not only patients with chronic pain (Bantick et al., 2002) but also those experiencing acute pain and experimental pain (Romero et al., 2013; Seminowicz & Davis, 2007; Sturgeon et al., 2015; Wiech et al., 2005). In turn, pain has also attracted the attention of scholars lately. Some EEG studies have shown that pain disrupts early neural potentials. The leading ERP index of deployment is the decrease of P3 amplitude that correlated with cognitive demand task and, thus, depicted a steal of attentional resources by pain (Houlihan et al., 2004; Samartin‐Veiga et al., 2019; Seminowicz & Davis, 2007). As revealed by Houlihan et al. (2004), P3 could be secondary to a slow wave, which shifts negatively under pain and is more evident in the more demanding condition. In this study, we also found the same “negative shift” of pain and in high load‐demanding condition. Moreover, this study revealed an interactive effect of pain and load in study participants. The amplitudes of early ERP components (N1, N2, and P2) exhibited a difference between pain subjects and painless subjects only in low loads. We observed no group difference in high cognitive tasks. Meanwhile, unlike the control group where subjects who can well inspect the load difference, those in the experimental pain group revealed no significant difference between loads in the early timing. In addition, N1, N2, and P2, the early components of ERP, are usually considered to primarily reflect the attention capture and arousal to stimuli (Peng & Tang, 2016). Perhaps, a smaller amplitude of N1 and N2 could indicate subjects’ lower involvement in the processing of the task stimuli (Huster, Enriquez‐Geppert, Lavallee, Falkenstein, & Herrmann, 2013; Samartin‐Veiga et al., 2019). Consequently, we speculated that experimental pain subjects could not attribute sufficient attention based on the cognitive demand unlike the healthy controls. As cognitive load increases, pain subjects might not use appropriate strategies to solve the task, assigning similar amounts of attentional resources to stimuli processing in both high and low cognitive tasks. Hence, we inferred that attention capture of pain subjects was insufficient or dysfunctional in the early phase, resulting in dysfunction in modulating attention based on the task demands.

We examined LPP by segments. LPP exhibited the same ERP significant difference as N1, P2, and N2 only in the 400–500 ms domain. That is, besides the same main effects, the control group also elicited significantly higher positive amplitudes than the experimental pain group in both high‐ and low‐load tasks, where no significance was noted again between loads in experimental pain subjects. Thus, we believed the early part of LPP (400–500 ms) suggested the same deficit in attention modification between loads, similar to N1, P2, and N2. For the late segments of LPP (500–800 ms), only the main effect (group and load) was constantly found. Usually, LPP amplitudes linked to task demands such as evaluation, memory encoding, and affect regulation (Zheng, Lyu, & Jackson, 2019). This study revealed that the LPP amplitudes were affected by not only loads, which was consistent with the research mentioned above, but also pain. Pain persons had lower LPP amplitude than those without pain, which could add a new understanding for this ERP component. Moreover, LPP had the highest amplitudes in the right hemisphere, followed by the central part and left hemisphere. However, no interactive effect was noted with group or other factors, suggesting that the hemisphere effect correlated with the paradigm we used in this study.

#### 4.2.2 Time–frequency analysis revealed a delayed peak energy for brain oscillation (delta and theta) in pain subjects and high‐load tasks

The time–frequency results in this study were also significant and had an overlap timing (500–800 ms) with LPP. With the frontal lobe as the main underlying region, the site of Fz revealed the most significant difference. Indeed, people rely on the frontal lobe for all types of abstract cognitive controls, including planning, memorizing, decision‐making, and higher‐level thinking, which are precisely the cognitive behavior involved in our tasks. Moreover, delta and theta oscillations exhibited a significant difference. Notably, we observed a trend that evoked the response of delta and theta later in high‐load than low‐load tasks, as well as in pain than that in nonpain persons. Pain, along with high cognitive load, had the latest energy peak. Perhaps, high load and pain inhibit neural oscillation, and this inhibition is time‐dependent that only occurs 500–800 ms after the onset, which is highly consistent with LPP in ERP results.

Delta oscillations often occur during deep sleep, but some studies have demonstrated that it also has a response with cognitive demand. In an ERP study of the Stroop task, Ergen et al. (2014) suggested a higher delta response involved in a relatively easier decision process when conducting a less demanding task. In addition, Selimbeyoglu, Keskin‐Ergen, and Demiralp (2012) revealed that the total delta was lower, especially in the uncertain response condition during a cognitive demand task. Başar, Schürmann, Demiralp, Başar‐Eroglu, and Ademoglu (2001) considered induced delta responses to be associated with stimulus assessment and decision‐making. Furthermore, delta oscillatory responses are reportedly different in certain crowds, like significantly lower in patients with MCI, compared with healthy controls (Güntekin, Emek‐Savaş, Kurt, Yener, & Başar, 2013). Consistent with the studies mentioned above, the observed delta oscillation across group and load denote alterations of the cortical networks that underlie cognitive controlling related to different conditions (group × load).

Event‐related oscillations in the theta frequency range correlate with central executive, attentional modulation, cognitive control, and working memory procedure (Cavanagh & Frank, 2014; Ishii et al., 2014; Sauseng, Klimesch, Schabus, & Doppelmayr, 2005; Tesche & Karhu, 2000). Like delta oscillations, theta oscillations in this study exhibited the same time‐dependent effect. Notably, theta oscillations in the EEG are primarily generated in the midline frontal brain areas, such as the medial prefrontal cortex and the anterior part of the midcingulate cortex (González‐Villar, Pidal‐Miranda, Arias, Rodríguez‐Salgado, & Carrillo‐de‐la‐Peña, 2017), which, perhaps, shares neural substrates for both pain perception and cognitive functioning. In addition, theta oscillations can be considered a neural signature supporting a coordinated response that suggests alertness, arousal, and/or readiness to process information (Başar, Başar‐Eroglu, Karakaş, & Schürmann, 2001). Painful stimulus and cognitive demand tasks can robustly activate regions in the prefrontal cortex and superior parietal lobes. In patients with chronic pain, the constant processing of pain could deplete neural resources that could be crucial to perform tasks that need attentional load, thereby affecting cognitive procedures (Samartin‐Veiga et al., 2019); this can also be applied to experimental pain subjects in this study, although the pain stimulus was not long. Thus, a late energy peak to pain feeling, together with cognitive load, suggested that the state of pain, as well as a high cognitive load, could cause a later procedure of information either because of early attention inhabitation or alertness delay.

### 4.3 Neural activities could illustrate the behavior of healthy persons under pain in different cognitive load tasks

Currently, several studies have focused on the attention deficit and corresponding brain function induced by chronic pain. For patients with chronic pain, excessive long‐term attention to pain not only causes impaired bottom‐up attention, which is manifested as difficulty in reorientation to information other than pain, but also impairs executive control of the individual's top‐down attention, making it impossible for the individual to shift attention to other goals than pain. The consensus is that the cognitive impairment of chronic pain primarily correlates with the default network dysfunction. Usually, the default network is activated in attentional activities, which include saline stimuli, or cognitive tasks, either decreasing deactivation (Baliki, Geha, Apkarian, & Chialvo, 2008) or strengthening the connection with other brain areas (e.g., insula) (Baliki, Mansour, Baria, & Apkarian, 2014; Napadow et al., 2010). However, the studies mentioned above mostly localized on the brain region and neural connection spatially but not temporally. Indeed, few studies focused on the mechanism of pain capture in healthy people. In studies including healthy subjects, pain appears as a distraction from attentional activity. Pain competes for attention, leading to a decreased activity in task‐related cortical areas. In this study, the application of ERP displayed a phenomenon of attention deprivation of pain in healthy subjects from the temporal aspect, by demonstrating an early attention deficient (difficulty in modulating attention based on the task demands in an early timing), as well as late attention inhabitation (a later oscillations of delta and theta because of pain or cognitive demand tasks). In addition, abnormalities in neural activities lead to behavioral abnormalities, resulting in a longer RT and lower AC in high cognitive load compared with low‐load tasks. Despite no difference in the behavioral RT between pain and painless subjects, the AC of pain subjects was lower because of the dysfunction of attention regulation. All these findings corroborated our original hypothesis.

### 4.4 Limitations

This study has several limitations worth acknowledging. First, although interfering words exerted no effect on experimental pain subjects in this study, it does not undercut the likelihood of an effect on a persistent painful state. In a chronic pain state, patients would experience a moderate level of pain for a prolonged period; this prolonged pain could also be accompanied by pain‐related fear and anxiety, not typically evoked in a controlled experimental setting. Thus, future research should include patients with chronic pain. Second, brain oscillations of the cortical processing of cognitive behavior were observed for experimental pain subjects under different cognitive loads; however, we could not localize the precise neural sources involved in these facilitatory effects because of low spatial resolution of the EEG technique. Perhaps, the EEG and functional magnetic resonance combination could be a better method to perform a comprehensive analysis. Finally, individual characteristics of pain could also play a role. Different individuals could have different attention strategies or attention habits when experiencing pain. Accordingly, brain mechanisms that regulate attention and pain could vary from individual to individual. Moreover, there could be discrepancy in brain mechanisms between “attention dominates” and “pain dominates.” Hence, subjects should be classified to determine the similarities and differences between different subgroups in future studies.

## 5 CONCLUSIONS

This study reveals that pain and cognitive load affect cognitive behavior, and early attention is inadequate in modulation according to the load difference because of pain. In addition, late attention is inhabited in a trend that energy peak for oscillations (delta and theta) was delayed under pain and in high cognitive load tasks. Overall, this study establishes a worse behavior in high‐load tasks and persons experiencing pain.

## DISCLOSURES

No conflicts of interest, financial or otherwise, are declared by the authors.

## AUTHOR CONTRIBUTIONS

Kangling Wang and Wen Wu contributed to the conception of the work. Kangling Wang, Yuqi Li, and Shimin Huang designed and collected the data. Guiyuan Cai and Rongdong Li analyzed the data. Kangling Wang wrote the article. All authors discussed the results and commented on the manuscript. All authors approved the final version of the manuscript.

## Data Availability

We confirm that our article contains a Data Availability Statement even if no data are available (list of sample statements) unless our article type does not require one. We confirm that we have included a citation for available data in our references section, unless our article type is exempt.
